# Aptamer Selection Technology and Recent Advances

**DOI:** 10.1038/mtna.2014.74

**Published:** 2015-01-13

**Authors:** Michael Blind, Michael Blank

**Affiliations:** 1 AptaIT GmbH, Munich, Germany

**Keywords:** aptamer, bioinformatics, microfluidics, next generation sequencing, NGS, SELEX

## Abstract

Over the last decade, aptamers have begun to find their way from basic research to diverse commercial applications. The development of diagnostics is even more widespread than clinical applications because aptamers do not have to be extensively modified to enhance their *in vivo* stability and pharmacokinetics in diagnostic assays. The increasing attention has propelled the technical progress of the *in vitro* selection technology (SELEX) to enhance the efficiency of developing aptamers for commercially interesting targets. This review highlights recent progress in the technical steps of a SELEX experiment with a focus on high-throughput next-generation sequencing and bioinformatics. Achievements have been made in the optimization of aptamer libraries, separation schemes, amplification of the selected libraries and the identification of aptamer sequences from enriched libraries.

## Introduction

Synthetic nucleic acid aptamers represent a unique class of high affinity ligands. They have demonstrated potential in applications ranging from therapy,^1,2,3^ targeted drug delivery,^4^ sensors^5^ to diagnostic reagents.^6,7^ In contrast to the biological processes used for the identification of protein ligands, aptamers are synthesized chemically and thus *in vitro* evolution can be used for their development and optimization.

**Figure 1:**
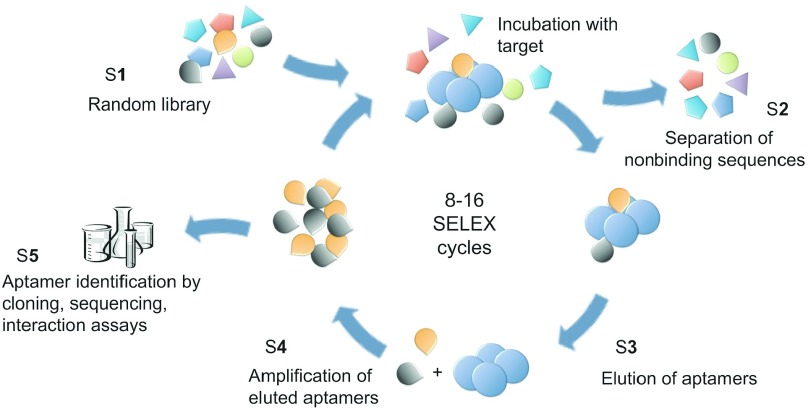
**The different steps (S1–S5) of the SELEX process.** The *in vitro* selection starts with random ssDNA or ssRNA libraries flanked by fixed primer annealing sites necessary for enzymatic amplification. The library is incubated with the target molecule and aptamer-target complexes are traditionally separated from nonbinding sequences by methods like nitrocellulose filter binding, electrophoretic gel mobility shifts or bead-based capture systems. The remaining nucleic acids are amplified by PCR in DNA aptamer selections or RT-PCR and RNA transcription in RNA aptamer selections. This selection cycle has to be repeated for 8–16 times to enrich the high affinity binding nucleic acids which are identified by cloning and Sanger sequencing. Starting-points for optimization: S1: optimized libraries, S2: better separation techniques, S3: alternative protocols, S4: omitted amplifications, S5: high-throughput sequencing and bioinformatic analysis.

The protocol for the *in vitro* selection of aptamers, SELEX, was developed in the year 1990 (refs. ^8,9^) and demonstrated the capacity of aptamers to bind a wide variety of target molecules ranging from large proteins to small molecules. The basic *in vitro* selection process is depicted in **Figure 1**. A conventional SELEX experiment needs several weeks to be completed manually. In recent years, the aptamer technology saw a significant upturn as novel developments in chemical synthesis, technical equipment, and analysis methods became available for enhancing the properties of aptamers and the efficiency of their development. A wide range of chemical modifications of the sugar phosphate backbone and the bases have been reported. Compared to the mere four natural nucleotide building blocks, these modifications either increase the stability in biological environments or increase the diversity of three-dimensional shapes and molecular recognition capacities.^10,11^ Significant progress was also achieved in optimizing the technical protocol of a SELEX experiment. An overview of the different steps (S1–S5) which have been improved is given in **Figure 1**. Here, we review recent developments to improve the SELEX process and to better understand the underlying evolutionary processes.

## Optimized Libraries for Aptamer Selection (S1)

### Analysis of starting libraries by high throughput sequencing

Originally developed for the purpose of whole genome sequencing and re-sequencing, next-generation sequencing (NGS) is changing the landscape also in many other areas of basic, applied and medical research.^12^ This trend can also be seen in the area of ligand discovery technologies like phage display^13,14,15^ and other geno-/phenotype-coupled technologies.^16^ NGS and subsequent computational data analysis are also applied on native as well as on enriched combinatorial libraries, opening new avenues in the field of aptamer identification and optimization.^17,18,19,20,21,22,23,24,25,26,27,28^

**Figure 2:**
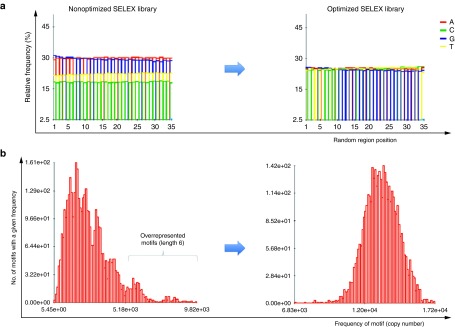
**Distributions of nucleotides and motifs of length six in a nonoptimized (left side) and a SELEX library which was optimized for equal nucleotide distribution (right side).** (**a**) The nonoptimized SELEX library (left side) is dominated by “A” and “G” and has a very unbalanced distribution of bases over all positions. After adjustment of the synthesis protocol, an almost even distribution of all four nucleotide bases (25% each) over all random positions was obtained (right side). (**b**) The distribution of motifs (length of six) in the nonoptimized library (left side) is unbalanced: Some motifs (on the side of high copy numbers) are highly overrepresented. After library optimization (right side) motifs of length six are equally distributed (Gaussian profile). Data were obtained by bioinformatic analysis with the software COMPAS at AptaIT GmbH.

NGS data derived from SELEX libraries can be used to perform a quality control on the basis of millions of sequences. The knowledge of the distribution of nucleotide bases over the positions of the random region allows to adjust the synthesis protocol and thereby an optimization of the respective SELEX library with respect to the desired distribution of nucleotides or motifs (**Figure 2**).

The design of the input library should provide an optimal starting point for the SELEX experiment. The most frequently used random region contains all four bases at an equal distribution. The rationale of many researchers is that short motifs within the aptamer sequence typically mediate binding to the target molecule. An equal distribution of motifs increases the sequence space and consequently the chance to end up with aptamers of the desired binding properties. Some alternative design strategies work with biased libraries which are a degenerate sequence of a known binding motif, for example randomized at a rate of 30% per position.^29^ This technique is useful to isolate mutated sequences which provide insights into the mode of interaction with the target or to improve binding characteristics. In any case, it is desirable that the library is conform with the specifications. Synthetic SELEX libraries do frequently not reach the required quality (**Figure 2**). NGS and bioinformatic analysis can thus help to closely monitor and optimize their synthesis.

### Reducing primer sites

A fixed constant in the SELEX process was the presence of primer annealing sites, which are used for the enzymatic amplification of the libraries after the separation steps. The primer sequences can contribute up to more than half of the aptamer sequence depending on the length of the random region. These design requirements of a SELEX library are in conflict with the desire to develop preferably short aptamer molecules which can be synthesized at an economic scale. Another reason for having less or no primer sequences is the concern that the constant sequences interact nonspecifically with the target or limit the complexity of the nucleic acid library by interacting with the random region. It is frequently observed that parts of the primers belong to the minimal binding motif and have at least some structural function.

Several protocols were developed which reduce the length of the annealing site or completely avoid the primer sequence. Previous attempts like tailored^30^ or dual SELEX^31^ still need residual conserved sequences of 7–10 nucleotides. The flanking regions in dual SELEX are sequestered by self-complimentary sequences minimizing the risk that they become part of the target-binding motif. More recent work has shown that it is possible to perform a SELEX experiment without any conserved nucleotides. The primer-free DNA aptamer selection^32^ was used to isolate aptamers against the target protein S100B with K_D_s in the range of 10^−7^ to 10^−8^ mol/l and employs endonucleases cleaving of the doubled stranded DNA template. The resulting library product has either two residual C at the 3'-end or no conserved nucleotides at all depending on the endonuclease used. The library is reconstituted after selection by ligation with primer annealing sites. This step is mediated by hybridization of a self bridge oligonucleotide. Related protocols have been used to isolate aptamers against HIV reverse transcriptase.^33^ The enzymatic reactions are more complicated than in a conventional SELEX protocol but provide an alternative when a primer-free design is desired.

## *In Vitro* Selection Methods (S2–S4)

### Acceleration of the *in vitro* selection process

One method for reducing the time required to perform an *in vitro* selection experiment is to vary the separation technique by omitting steps from the traditional protocol. The RAPID process (RNA aptamer isolation via dual cycles) shortens the time required by performing consecutively two binding and separation steps without amplifying the selected RNA libraries by reverse transcription, PCR and transcription.^34^ An *in vitro* selection against two target proteins, UBLCP1 and CHK2, compared a conventional *in vitro* selection with six cycles and amplification after every separation with RAPID where six separations but only three amplification steps were applied. The time to complete the experiment decreased from 355 hours to 84 hours. Interestingly, both experiments resulted in the enrichment of identical sequences in the top five candidates. RAPID provides therefore a fast alternative for *in vitro* selections with the advantage that it can be integrated into routine selection protocols without the need to change parameters or equipment.

Similarly to RAPID, which relies in principle on a more effective separation of binding from nonbinding sequences, the processing time for aptamer identification has been reduced by more effective separation equipment. Capillary electrophoresis based SELEX or CE-SELEX has been used for the isolation of a number of specific aptamer sequences for protein targets.^35,36,37,38^ Its advantage is that the very efficient separation of free nucleic acids from aptamer/target complexes reduces the number of cycles needed for the isolation of high-affinity aptamers to four or sometimes even less. Recently, CE-SELEX was also applied to the small molecule N-methyl mesoporphyrin (NMM), which is more challenging since a good resolution of complexes and free oligonucleotides is more difficult due to the minimal differences in the mobility shift.^39^ Advanced material like the boronate affinity monolithic capillary, which is a very effective capture/release matrix for glycoproteins, can be used to enhance the efficiency of CE-SELEX for defined target classes.^40^

Another CE-method, which was called Non-SELEX, applies nonequilibrium capillary electrophoresis of equilibrium mixtures (NECEEM) for partitioning.^41,42,43,44^ Only three rounds of separation without intermediate amplification were necessary to improve the affinity of a DNA library to a target protein by more than four orders of magnitude. Time required to complete the experiment was just one hour. MonoLex is a related protocol which includes just one chromatographic separation step and no consecutive *in vitro* selection cycles.^45,46^

However, it has to be considered that electrophoretic mobilities of aptamer-target complexes and the bulk of unbound nucleic acids, not only depend on the size and charge of target molecules but also on the applied buffer system. The low ionic strength and pH have to be in line with the assay system for which the aptamer is developed. These conditions are sometimes in conflict with CE-SELEX. Moreover, CE-SELEX allows only the application of very low reaction volumes restricting the overall amount of the starting library. Above all, both the salt concentrations as well as the temperature continuously shift in the course of an electrophoresis run. Consequently, the aptamers final binding conditions are difficult to adjust.

A similar approach applies affinity chromatography but circumvents the problem of small reaction volumes. A microfluidic free flow electrophoresis (μFFE) device was used to select DNA aptamers for human immunoglobulin E (IgE). μFFE allows to load ~300-fold more library (10^14^ molecules) through a continuous flow if compared to capillary electrophoresis based selections. Aptamers with low nmol/l dissociation constants for IgE could be identified already after one selection cycle, indicating that μFFE is a very efficient separation method.^47^

### Fully integrated *in vitro* selection—SELEX-on-a-chip

SELEX is an *in vitro* selection process, which is conducted by applying very defined protocols and quite robust enzymatic amplification. Furthermore, it does not require any amplification in biological systems. Aptamer selection was consequently automated on robotic pipetting systems in 96 well formats, allowing a massive parallelization of aptamer discovery.^48,49,50^

Additionally, SELEX is perfectly suited to be integrated in microfluidic chip based systems. The immobilization of targets on magnetic beads is a very common *in vitro* selection method since it enables easy separation of bound nucleic acids by applying a magnetic field and washing away the rest of the nonbound library. The magnetic capture can be miniaturized in microfluidic channels also referred to as M-SELEX. Micro magnetic separation (MMS) was applied to evolve aptamers which bind the target proteins streptavidin, botulinum neurotoxin type A and PDGF-BB with K_D_s in the low nanomolar range.^17,19,51,52,53^ Tuning the stringency conditions resulted in significantly higher affinities for the targets PDGF-BB and thrombin with respective dissociation constants of 0.028 and 0.33 nmol/l.^54^

Proceeding to the next level, a fully integrated chip-based *in vitro* selection platform, based on magnetic separation of target coupled magnetic beads on a continuous-flow magnetic separation device was developed to screen aptamers automatically against the protein targets CRP^55^ and AFP.^56^ The miniaturized set-up integrates magnetic separation, micropumps, micromixers and temperature control systems for enzymatic amplification reactions. The integrated discovery platform was also successfully applied to discover aptamers against a complex target, the influenza A/H1N1 (InfA/H1N1) virus.^57^

### Alternative separation strategies

The target of *in vitro* selection procedures is usually immobilized to allow the separation of bound aptamers from the bulk of unbound nucleic acid sequences. Isolation of the aptamer/target complexes can also be achieved by size or charge mediated separation, *e.g.*, gel shift or gel filtration. These strategies are difficult to apply to small molecules since there is little difference in the molecular weight or charge of the complex compared to the aptamer alone. Chemical immobilization on surfaces is on the other hand often difficult due to the lack of suitable functional groups or the significant physical changes of the small molecule due to the chemical modification. Stoltenburg *et al*. proposed an alternative separation schedule, Capture-SELEX, where the nucleic acid library is immobilized on magnetic beads and aptamers are eluted by specific binding to the target.^58^ A library was designed which contains a short fixed hybridization sequence, a so called docking sequence, located between two random regions. The library is captured on magnetic beads via a complimentary strand to the docking sequence. Aptamers are specifically eluted from the beads by addition of an interacting small molecule target. During the binding reaction, they undergo a specific conformational change and detach from the docking sequence. Specific aptamers for the antibiotic Kanamycin A, which bind with low micromolar affinitiies were selected in a first proof of concept study. The use of the docking sequence provides another advantage. The evolving aptamers have already a functional displacement system, the docking sequence, which can be used to design various reporter systems based for example on fluorescent beacons, fluorescence resonance energy transfer or fluorescence polarization. No laborious post-SELEX modification is therefore necessary. Bead-based or MTP-based fluorescence detection assays were consequently used to characterize the binding characteristics of the anti-Kanamycin A aptamers and to demonstrate the straightforward assay development with Capture-SELEX derived aptamers.^59^

## Identification of Aptamers by Next Generation Sequencing and Bioinformatic Analysis (S5)

### Identification at high resolution

The conventional SELEX-approach of cloning, colony picking and Sanger sequencing of a small number of colonies typically gives access to the most frequent clones. Identical sequences are counted and subsequently aligned in order to group similar sequences. By usage of NGS, a quite similar procedure is typically applied: the giant raw data sets are analyzed in terms of counting sequences and ranking them in the order of frequencies.^19,21,25,26,27^ Sequence frequencies can be analyzed at very high resolution. Sequences can be ranked and filtered by a cut-off of read numbers to select candidate aptamers for further testing.

### Identification in early selection cycles

SELEX is typically a laborious process. A major goal is the identification of aptamer ligands with a minimal number of SELEX cycles. In addition to economic reasons like costs of reagents and time, there are further arguments for aptamer identification in an early selection round. The SELEX process is accompanied by extensive enzymatic amplification steps. It is obvious that in the course of a multicyclic SELEX experiment the evolution pressure shifts from the side of high affinity sequences to sequences which are just better amplifying.^19,20^ This amplification bias favors shorter as well as structurally less stable sequences.^28^

Unfortunately, early identification of aptamers is not routinely possible by applying NGS and simple sequence counting. In addition to an efficient separation step to remove background binders, more sophisticated bioinformatic approaches have to be applied. Kapakuwana *et al*.^20^ focused on aptamer selections using so-called over-represented libraries which had only 15 nt long random regions (embedded in a conserved stem structure). Copy counts in the range of thousands for individual sequences are statistically present in 100–500 pmol input material of such relatively short starting libraries. Enrichment is consequently easier to detect as aptamers are already present in higher copy numbers after the first SELEX steps. Antithrombin aptamers were identified by performing only one cycle of selection, separation and amplification followed by high-throughput sequencing and counting of full-length sequences. The most dominant clone was the same aptamer already identified by Bock *et al*.^60^

However, starting libraries with shorter random regions cover just a restricted sequence space due to the higher copy number of individual sequences and the disproportionally high contribution of primers in aptamer structure formation. It can be expected, that screening of more diverse libraries with longer random region lengths (≥30 nt) will routinely result in the better aptamers. Like Kupakuwana *et al*.^20^ also Hoon *et al*.^24^ managed to identify antithrombin aptamers in a single selection cycle by applying NGS and *in silico* analysis, but with the difference that standard length SELEX libraries were applied. Aptamers were identified by focusing on frequent subsequences (also referred to as motifs or k-mers) within the 33 nt long random part of the applied “hairpin-structured” library. Over-represented subsequences provide strong evidence that the respective full-length sequence had an advantage during the selection process and thus might be a promising candidate for testing.^18,61^ Full-length sequences were truncated to the identified 15–16 nt long motifs and tested for binding to the target thrombin. Nine of 10 subsequences (among them the high affinity aptamer identified by Bock *et al*.^60^) were positive. However, it cannot be assumed that truncation to subsequence length always maintains the sequence binding capability since subsequences typically only function in the context of its full-length sequence only.

However, just considering subsequence frequencies might be misleading. Motifs in starting or enriched libraries are typically not equally distributed due to a synthesis bias. Therefore, the factor by which a motif is being enriched from one library to another (also termed amplification fold value), gives an additional measure for its or the respective full-length sequence relevance.^19,21,62^

### Representative selection of aptamer candidates for interaction assays—clustering of sequences

The most potent sequences are not necessarily those with high frequencies. Underrepresented sequences might be the aptamers with the most preferred properties. NGS and *in silico* analysis typically yield so many potential aptamer sequences that deconvolution of sequences gets absolutely mandatory.

The deconvolution of sequences is typically achieved by clustering of related sequences. The clustering of identified sequences is performed independently from their copy number. In the next step, sequences from the individual clusters can be “picked” as representatives for each cluster. In doing so, a high number of unrelated sequences, which presumably cover the maximum amount of different binding epitopes on the target protein can be tested in functional assays. Once an active cluster has been identified, analysis and testing can be performed in this more focused sequence space.

Ditzler *et al*.^18^ identified individual evolutionary lineages within SELEX populations selected to bind HIV-1 RT by clustering of sequences based on conserved motifs and structural similarity. The comparison of NGS data within and between clusters allowed the identification of highly potent aptamer inhibitors that block primer extension by the RT of HIV-1 and inhibit HIV-1 replication in cells very effectively. The fact that the most potent aptamer was very rare (0.4% of the enriched population) underlines the usefulness of the applied analysis schedule that clusters, aligns, and compares sequences based on patterns of conservation and covariance.

In other groups, clustering was also performed on the basis of secondary structure to successfully identify aptamers binding to norovirus capsid protein VP1.^61^ Also antineurotropin receptor TrkB^27^ as well as vascular smooth muscle cells (VSMCs) internalizing aptamers^62^ could be identified by secondary structure guided approaches.

### Tracing aptamer populations

Another criterion to select defined sequences for binding or functional analysis can be the increasing or decreasing trend of sequence populations.^62^ Schütze *et al*.^21^ analyzed the dynamics of specific binders during the selection process by tracking their frequency over the successive SELEX rounds. Aptamers with different enrichment behaviors could be identified: some sequences dominated early selection cycles whereas other sequences appeared in later cycles and dominated the finally enriched pools. Other sequence populations continuously increased in the course of the SELEX experiment. Such tracking results make it possible to compare individual aptamers with respect to affinity, amplification behavior or competition for binding to the same target epitope.

**Figure 3:**
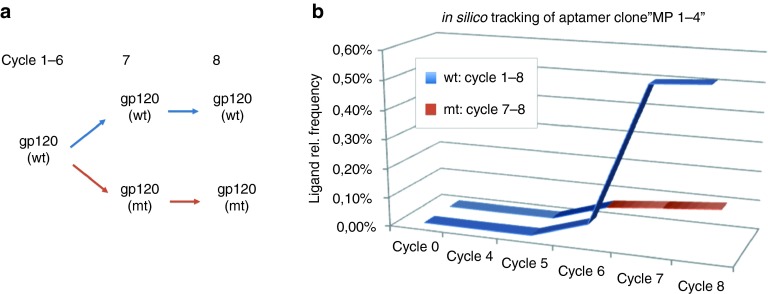
**Strategy for the identification of aptamers addressing a defined target epitope.** (**a**) Selection scheme: SELEX was performed for six rounds against the wild-type (wt) form of recombinant gp120. After the sixth cycle, the selection was branched: SELEX was performed for two additional cycles against the wt-protein (blue path). SELEX cycles 7 and 8 were performed in parallel against a variant of gp120, which was mutated at the binding site of interest (red path, gp120 mt). (**b**) *In silico* tracing of aptamers on a monoclonal level revealed one rare candidate (clone MP 1–4), that was enriched to about 0.5% in the finally enriched SELEX round (blue curve). Tracing of aptamer MP1-4 over NGS data derived from the cycles 7 and 8 from the control SELEX experiment revealed significant lower enrichment (red curve).

The method of tracing the frequency of sub- or full-length sequences over different selection rounds can also be used to identify clones addressing defined binding sites on the target protein. SELEX experiments can be performed as control to check the occurrence of potential aptamer candidates in SELEX experiments where respective aptamers are expected to be present or absent. **Figure 3** describes a strategy, which enabled the identification of an aptamer addressing a defined epitope on the target protein gp120 (data from AptaIT GmbH). A SELEX control was performed against a variant of gp120 mutated at the binding site of interest. Specific binding of the aptamer clone “MP 1–4” to the wild-type protein was confirmed after its synthesis and testing in filter retention assays.

### Aptamer optimization

Once an aptamer with desired binding properties has been identified, more detailed analysis of the sequences within the cluster of the “winner sequence” can be performed. This high-resolution perspective by NGS data set analysis consequently boosts the laborious post-SELEX aptamer optimization phase. Sequences which are members of the same clusters or families can be analyzed using alignment based tools^63,64^ or tools based on secondary structures predictions.^65,66,67,68^ In doing so, essential sequence features and structural motifs like protruding stems and loop regions that are determinants of aptamer affinity can be identified.^18,27,61,62^

Analysis of NGS data belonging to the sequence space of the relevant functional aptamer ligand, enabled Ditzler *et al*. to identify essential structural motifs within this aptamer population.^18^ A series of software tools was applied within a precisely composed analysis pipeline: software and scripts for filtering the raw sequence data and for clustering and alignment of closely related sequences had to be used. Another software to predict secondary structures based on conservation and covariation of sequences within individual clusters was applied for the identification of structural motifs. The covariation models based on the provisional secondary structures and alignment was created with an additional software tool. This makes clear that expert knowledge in the field of SELEX as well as in the field of bioinformatics is necessary to efficiently harness NGS to leverage aptamer identification and optimization.^18,61,62^

## Discussion

Despite recent promising therapeutic^3,69^ and diagnostic^70,71,72^ developments, a bottleneck is still the discovery of high quality aptamers against relevant targets. Consequently, technological progress was implemented in the SELEX process to enhance the performance of aptamer discovery. NGS and bioinformatic analysis were used to optimize the synthesis protocols of synthetic nucleic acid libraries in terms of a better balanced nucleotide and motif distribution to ensure (i) a maximal diversity and target binding capacity and (ii) to repress the influence of the constant primer annealing sites. A further step forward is the improvement of the *in vitro* selection process itself by reducing the time required to complete the aptamer selection by improved separation techniques ideally combined with a miniaturization of the equipment. Automatic chip-based *in vitro* selection platforms are technically challenging but a promising approach that provides several advantages: once established, the automatic procedure reduces labor, reagent consumption and overall time needed to complete aptamer selection experiments.

Sequencing technology in high-throughput formats and computational analysis of SELEX experiments also improve aptamer discovery. Hundreds of millions of sequence reads are now routinely generated with the desired length and quality. Molecular indexing will be a strategy to combine multiple SELEX populations in a sequencing reaction, thus lowering sequencing costs per SELEX experiment. Therefore, cost will be no longer a barrier to the use of NGS in SELEX experiments in the future. A great challenge for the future is the combination of different achievements and their integration in one platform. The power of a combination between SELEX on a chip, subsequent NGS and multiparallel interaction studies has for example been demonstrated in the QPASS System.^17^

A high throughput SELEX process needs to be fed with a large number of targets. Provision of enough proteins for example is a challenging task since they have to be in a correct three-dimensional folding state and posttranslationally processed. Bacterial or yeast expression systems often produce misfolded proteins and do not bear mammalian, posttranslational modifications. So-called Cell-SELEX employs cells bearing the correct target in a cellular context in the selection process and helps to resolve some problems with difficult targets.^73,74^ Computational analysis to extract the relevant information from the giant sequencing datasets is another hurdle. The expertise to combine multiple bioinformatic tools and develop stand-alone solutions^18^ is not available in most working groups. To solve this problem, we have developed the software COMPAS (COMmon PAtternS) which has all essential features integrated.^75^ COMPAS enables straightforward quality control of starting libraries (**Figure 2**) and aptamer identification in early selection cycles by sophisticated clustering of sequences based on covariance models of frequent motifs. An efficient identification and deconvolution of aptamers for functional tests can be assured in combination with an integrated feature, which enables tracking the frequency of full-length– and subsequences over subsequent selection rounds. Easy to use software tools like COMPAS provide a route to make the high-throughput sequencing technology accessible for SELEX labs without bioinformatic expertise.

More interdisciplinary work will be necessary to develop fully integrated solutions. Close monitoring of the binding behavior of the enriched libraries, feedback to optimize the selection conditions, and preparation of sequencing templates will be implemented in the same platforms enabling a fully automated, experimental platform in the future.
